# Some Bioactive Natural Products from Diatoms: Structures, Biosyntheses, Biological Roles, and Properties: 2015–2025

**DOI:** 10.3390/md24010023

**Published:** 2026-01-04

**Authors:** Valentin A. Stonik, Inna V. Stonik

**Affiliations:** 1 Laboratory of Chemistry of Marine Natural Products, G.B. Elyakov Pacific Institute of Bioorganic Chemistry, Far Eastern Branch, Russian Academy of Sciences, Pr. 100-Letya Vladivostoka 159, Vladivostok 690022, Russia; stonik@piboc.dvo.ru; 2 Laboratory of Marine Microbiota, A.V. Zhirmunsky National Scientific Center of Marine Biology, Far Eastern Branch, Russian Academy of Sciences, ul. Palchevskogo 17, Vladivostok 690041, Russia

**Keywords:** diatoms, pigments, carbohydrates, lipids, oxylipins, sterols, isoprenoids, toxins, miscellaneous compounds

## Abstract

Recently, as a result of growing interest in diatoms as sources of energy (biofuel) and valuable food components for humans and aquaculture organisms, new data on the structures and properties of diatom natural products have been obtained, including both endo- and exometabolites. Information about their biosynthesis, biological activity and roles, and their beneficial and hazardous properties has also emerged. The application of modern methods of molecular biology, metabolomics, and chemical ecology to the study of diatom natural products has improved the understanding of many important natural phenomena associated with diatoms, such as photosynthesis, harmful algal blooms, interactions of diatoms with other organisms of marine biota, and their impact on biogeochemical cycles and climate regulation. In this paper, we discuss various aspects of research on natural compounds from diatoms, covering the last decade, as well as prospects for their further development, which have become apparent in recent years.

## 1. Introduction

Diatoms are an important group of aquatic microorganisms that produce a wide range of bioactive, mainly low-molecular-weight natural products. Ten years ago, we reviewed structures, taxonomic distribution, and biological role of such metabolites [1]. 

Diatoms represent either single-cellular or colonial microscopic low plants of a great diversity and significance, which are suggested to have appeared in the early Jurassic period at least 185 million years ago, although sedimentary evidence suggests much earlier origin. Diatoms became the major plankton constituents in the ocean until the Cretaceous, when the breakup of the supercontinent Pangaea caused the formation of major ocean basins, accompanied by the increased delivery of nutrients in the oceans. Usually, planktonic diatoms live as suspended microorganisms in the sunlit surface layer of seawaters and are moved by winds and sea currents.

The contribution of diatoms to various biogeochemical cycles facilitates the intake of carbon and silicon into the marine environment, followed by the removal of excess of CO_2_ and organic matter from seawaters and the sequestering of carbon in bottom sediments [2,3]. Although they represent only 0.2% of the Earth’s photosynthetic biomass [4], diatoms are the most important producers of primary food for other marine organisms and form the basis of their food chains. Diatoms also contributed to the formation of fossil fuels such as petroleum and gas [5] and participated in global CO_2_ sequestration from the atmosphere and seawaters [6]. A great genetic diversity of diatoms in the World’s Oceans was confirmed, using collections of Tara Oceans expeditions, which took place from 2009 to 2013 aboard the schooner (Tara). As a result, a tremendous number of new genes were found in these microalgae [7]. Moreover, diatoms are widely distributed not only in marine, but in other aqueous environments, including freshwaters, hot springs [8], and moist soils. Some of these microalgae are known as endosymbionts or epiphytes of other microalgae, for example, dinoflagellates, where they may be present instead of peridinin chloroplasts [9]. Sometimes, multicellular macroalgae and marine invertebrates also contain symbiont diatoms [10,11].

The origin of diatoms, which have four membranes in chloroplasts, was the result of secondary symbiosis between a heterotrophic eukaryote and a red alga, unlike the majority of other photosynthetic organisms with a two-layered plastid membrane, which appeared as a result of primary symbiosis. Chloroplasts of diatoms provide more effective oxygenic photosynthesis than those in other microalgae [12].

Nowadays, these low plants present the most successful and species-rich group of microalgae with a number of actually existing diatom species, estimated at least from 30,000 up to 100,000 [13]. Traditionally, for many years, diatoms were divided into subgroups of radial symmetric centric and pennate (araphid and raphid) diatoms, the latter being bilaterally symmetric. However, at the beginning of this century, classification of diatoms received a new development, and several subphyla with three classes of diatoms, including Coscinodiscophyceae (radial symmetric centric diatoms), Mediophyceae (polar centric diatoms with bi- and multipolar symmetric valves and radial symmetric Thalassiosirales), and Bacillariophyceae (pennate diatoms), were established [14,15].

The recent progress in diatom genomics has led to about a dozen whole genome sequences established, and has shown that these microalgae are particularly interesting as model organisms to better understand peculiarities of eukaryotic evolution [16].

The diversity of diatoms and their capability to adapt to environmental changes is closely related to the variety of natural products biosynthesized in these microorganisms. Herein, we discuss results regarding some groups of biologically and ecologically active natural products from diatoms over the last decade. This review covers a significant portion of recent data in this field. Recently, several interesting reviews have also been published that broaden the scope of this paper, which is important for understanding various aspects of diatom metabolite studies; see, for example, [17,18,19,20].

When writing this paper, we conventionally classified diatom metabolites into endo- and exometabolites, although we were aware that this division is somewhat arbitrary. This is particularly true for microorganisms such as diatoms that form the basis of many food chains. Nevertheless, the chosen framework helped us to present and discuss the extensive data published in the last decade.

The purpose of this review is to illustrate how knowledge about the structural diversity, biosynthesis, and beneficial properties of certain notable groups of diatom metabolites have evolved over the past decade.

## 2. Endometabolites from Diatoms

### 2.1. Pigments

#### 2.1.1. Fucoxanthin (Fcx)

**Scheme 1:**
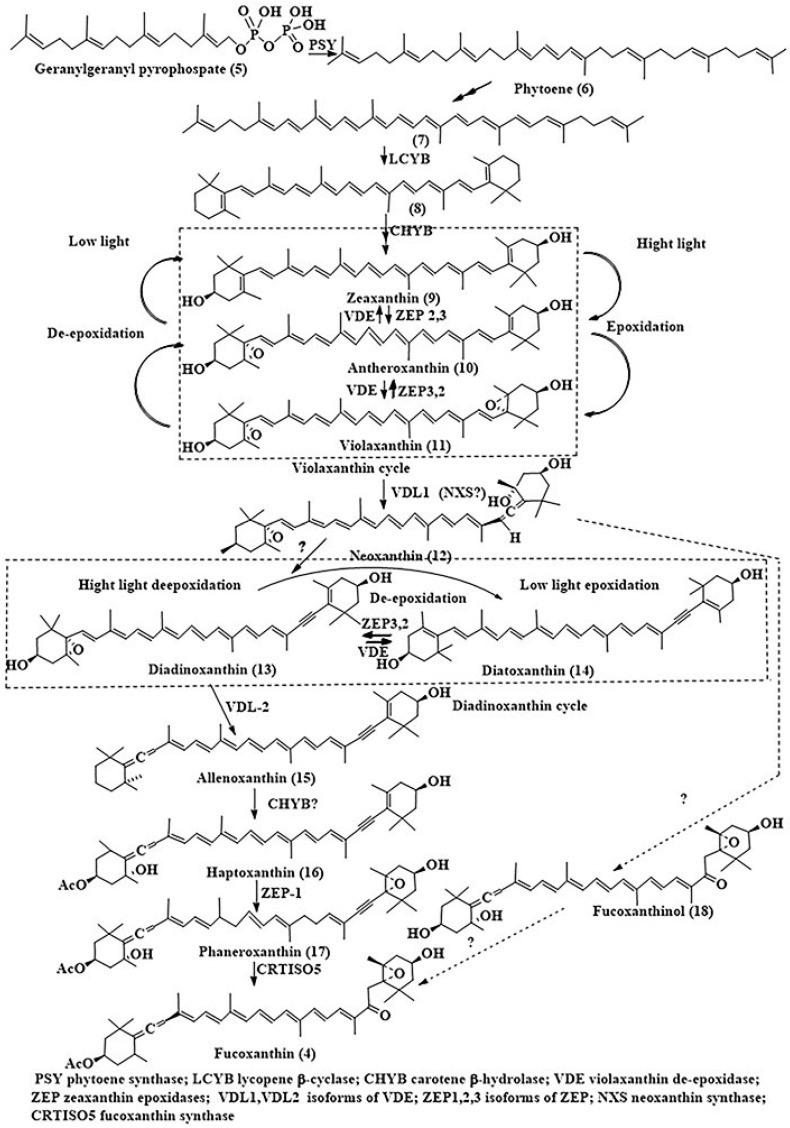
The scheme of fucoxanthin biosynthesis, adapted from [22,23,24,26,27]. PSY—phytoene synthase; LCYB—lycopene β-cyclase; CHYB—carotene β-hydrolase; VDE—violaxanthin de-epoxidase; ZEP—zeaxanthin epoxidases; VDL1, VDL2—isoforms of VDE; ZEP1,2,3—isoforms of ZEP; NXS—neoxanthin synthase; CRTISO5—carotenoid isomerase 5.

**Figure 1:**
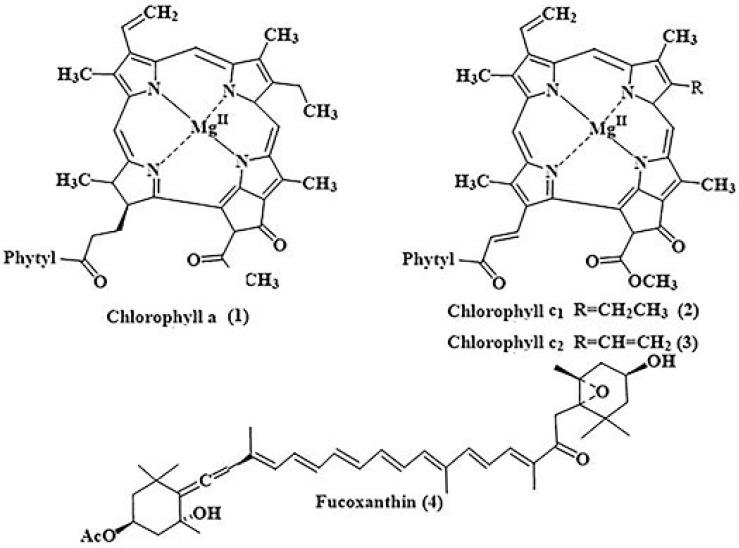
Main pigments, taking part in photosynthesis in diatoms.

Some pigments are responsible for capturing solar energy for photosynthesis to provide oxygen production and organic matter synthesis and accumulation. A group of light-harvesting pigments in diatoms includes chlorophyll a (**1**), chlorophylls c1 (**2**) and c2 (**3**), and the carotenoid pigment fucoxanthin (Fcx, **4**), as well as some minor derivatives of chlorophylls and fucoxanthin-related pigments (Figure 1). Carotenoids (not less than 850 different natural compounds) are the most widely distributed natural pigments, which are found in all taxa of plants, as well as bacteria, fungi, and many animals. Numerous carotenoid pigments, including those found in diatoms, are biosynthesized from active forms of isoprene units to yield geranylgeranyl pyrophosphate (**5**). This leads, through intermediate lycopene (**6**) and β-carotene (**7**), to a series of bioactive oxidized derivatives (xanthophylls), for example, to **8**–**18** (Scheme 1). Carotenoids carry out not only structural function, being important constituents of plastids, but also play two more important biological roles, essential for diatom cell life. They provide photosynthesis and photoprotection against the formation of active oxygen species, induced by light excess. The photosynthetic function of diatom carotenoids is implemented mainly by fucoxanthin (Fcx, **4**) and some closely related xanthophylls [21]. Pigments (**1**–**4**) form complexes with special proteins of chloroplasts and participate in the absorption of blue-green light energy, penetrating to deeper water, to transfer the energy into the systems of photosynthesis or to disperse the surplus energy as heat. Wang et al. [22] have established the structure of a dimeric fucoxanthin chlorophyll binding protein (FCP) from the diatom *Phaeodactylum tricornutum* using X-ray analysis. Its crystal structure showed that this complex attaches seven molecules of chlorophylls a, two chlorophylls c, seven fucoxanthins, and probably one diadinoxanthin within the protein scaffold. Therefore, they established that the FCP is structurally related to the light-harvesting complexes of brown macroalgae and higher plants, but have more binding sites for xanthophylls and fewer for chlorophylls in comparison with the complexes in other plants. In contrast with terrestrial higher plants, which use mainly chlorophylls as light–harvesting pigments, brown-colored algae including diatoms, haptophytes, and brown macrophytes also employ fucoxanthin (Fcx, **4**) for this purpose [22].

Structurally, (Fcx) is unprecedented when compared with other carotenoids. Its molecule contains two structurally unusual termini, one having the allenic fragment, rare in natural products and linked to acetylated β-ionol ring, and another composed of epoxidated β-ionol ring, bound with the CH_2_CO group. Fucoxanthin belongs to rare oxidized carotenoids, containing six oxygen atoms [21]. This compound is one of the most abundant carotenoids in the world’s oceans. Its content in diatoms was established to be approximately 1–6% of the dry weight, which is about 100 times higher than in brown macrophytes, another known source of Fcx [23].

Diatoms can be considered as cell factories for the production of this high-value product, possessing properties valuable for human health, which may be used not only in nutrition, for example, in the feeding of broiler chicken, and cosmetics, but also as new pharmaceutical compositions and food additives [23]. Traditional production of Fcx from brown macroalgae is constrained by low yields and high cost. The purified pigment is very expensive at present; its prices have reached up to 80,000 USD/kg in 2016 with more than 500 t produced annually and the 5–6% increase in the following years. So far, the industrial production of this carotenoid pigment by diatoms is not yet sufficiently cost-effective to compete with that based on other approaches [23,24].

However, the studies on biosynthesis of **4** in diatoms and improvement in cultivation and extraction methods for the target product open the perspective of its acquisition on a large scale in controlled bioreactors using selected or even genetically modified strains and applying methods of biotechnology and synthetic biology for its production [25].

Recently, the diatom *P. tricornutum* as an easily and rapidly cultivated species with a known whole genome sequence has become a convenient microalgal model for studying the biosynthesis of Fcx. Like other carotenoids in different organisms, Fcx of diatoms is biosynthesized mainly by transformations of geranylgeranyl pyrophosphate (**5**), a product of the telomerization of three isopentyl and one dimethylallyl pyrophosphate molecules. Two molecules of **5** are coupled to each other in the “tail to tail” manner followed by conversions into trans-phytoene (**6**), lycopene (**7**), and β-carotene (**8**), for review of this part of Fcx biosynthesis [26]. Further transformations of **8** lead to a series of Fcx precursors such as zeaxanthin (**9**), antheroxanthin (**10**), and violaxanthin (**11**), participating in the so-called violaxanthin cycle. This cycle regulates the redistribution of light energy between violaxanthin, zeaxanthin, and chlorophyll a. Zeaxanthin (**9**), being a biosynthetic precursor of Fcx, simultaneously plays the phytoprotective role in the dissipation of excessively absorbed light energy as heat by non-photochemical quenching. In contrast, violaxanthin (**11**) as a molecular antenna collects photons and transfers them to chlorophyll a, facilitating photosynthesis. The reversible conversions of **9** into **10** and then **11** and vice versa are catalyzed in plants by zeaxanthin epoxidases (ZEPs) and violaxanthin de-epoxidases (VDEs).

In diatoms and other chromalveolate algae, such enzymes are represented by isoforms of VDE and ZEP, which appeared as a result of duplication of the corresponding ancient genes and were triggered during evolutionary search for the expansion of carotenoid functions from photoprotection to effective light harvesting. Some of them were named violaxanthin de-epoxidase–like proteins (VDLs). They are closely related to the VDE of higher plants and algal macrophytes and involved into the biosynthesis of Fcx and some other xanthophylls [27].

Xanthophylls (**9**–**11**) were found in the concentrated carotenoid fraction of the diatom *P. tricornutum* as well as one more intermediate, known as neoxanthin (**12**). Neoxanthin was supposed to be a branching point on the pathway to Fcx, converting into both diadinosterol and into Fcx itself bypassing the diadinoxanthin stage. The neoxanthin synthase (NXS) gene, which was not previously annotated in the diatom genome, was recently cloned and studied. However, the relation of this gene to violaxanthin de-epoxidation enzymes was not shown [28]. Later, Li et al. [29] compared pigments of 10 different *P. tricornutum* strains, collected in globally different locations, and found that one of the strains exhibited the highest content of Fcx fucoxanthin, but had a low biomass. Transcriptome analysis revealed that the high content of this pigment in the Pt6 strain is due to the higher expression of the VDL1 gene, which encodes the enzyme and catalyzes the conversion of violaxanthin to neoxanthin in the biosynthesis of Fcx.

Previously, Bai et al. [30] identified new paralogs (genes separated by gene duplication and neofunctionalization events) of VDE and ZEP as the corresponding key enzymes of fucoxanthin biosynthesis. For example, violaxanthin de-epoxidase-2 (VDL2) and zeaxanthin epoxidase (ZEP1) were shown to be involved in the biosynthesis of Fcx in the *P. tricornutum*. Knock-out of these genes, encoding the formation of xanthophylls pigments in the diatom, converts the algae into green due to the lack of Fcx. The scheme of Fcx biosynthesis was proposed with diadinoxanthin (**13**), which is reversibly converted into diatoxanthin (**14**) in the duadinoxanthin cycle as well as into allenoxanthin (**15**), haptaxanthin (**16**), and phaneroxanthin (**17**) as the following intermediates in the biosynthesis of Fcx (**4)**. Thus, the repeated epoxidation and de-epoxidation take place in both violaxanthin and diadinoxanthin cycles, participating in both photoprotection and the biosynthesis of Fcx. Diadinoxanthin is converted to allenoxanthin by violaxanthin de-epoxidase-like 2 (VDL2), whose function is confirmed using a green mutant of *P. tricornutum*.

Further efforts by Cao et al. [31] established the final step of the pathway, as catalyzed by carotenoid isomerase-like protein 5 (CRTISO5). Surprisingly, instead of serving as an isomerase, in diatoms, this enzyme catalyzes the hydration of a carbon–carbon triple bond within the precursor molecule (**17**) that leads to the appearance of α, β-unsaturated ketone moiety in Fcx (so-called ketolation stage of the biosynthesis). CRTISO proteins were earlier known as isomerases which catalyze C=C double bond isomerization in isopenoids of cyanobacteria and terrestrial plants. Mutation of CRTISO, leading to CRTISO5 in *P. tricornutum*, turned the diatom algae green with the overaccumulation of its substrate, phaneroxanthin (**17**). The change in producer color to green is typical for the loss of Fcx synthesis. It was suggested that CRTISO5 hydratase activity evolved via gene duplication and neofunctionalization. This discovery opened up new possibilities for the use of biocatalytic production of Fcx for the needs of healthcare [32].

The clarified total scheme of Fcx biosynthesis in *P. tricornutum* was recently proposed by Giossi et al. [33], who summarized that zeaxanthin epoxidases and violaxanthin de-epoxidases are represented in the diatom by different isoforms such as ZEP1, ZEP2, ZEP3 and VDL1, VDL2, participating in biosynthesis of Fcx (**4**). At that, VDL1 and VDL2 are involved in the biosynthesis, VDE and ZEP3 orchestrate photoprotective xanthophyll cycling, while ZEP2 proved to be an interface in this diatom between photoprotection and de novo biosynthesis of Fcx (**4**) [33]. In addition, the conversion of neoxanthin (**12**) into Fcx (**4**) was suggested in the recent review by Takaichi [34] to proceed through the intermediate fucoxanthinol (**18**), which was previously known as a metabolite of Fcx (**4**) in its consumers. Not all the stages of Fcx biosynthesis in diatoms (Scheme 1) have been identified to date, for example, the enzymes which catalyze conversion of neoxanthin (**12**) to diadinoxanthin (**13**), as well as those introducing an acetate group into haptoxanthin (**16**) and fucoxanthinol (**18**), remain unknown.

Although data on the structural diversity of carotenoid pigments in diatoms have not expanded significantly, understanding the individual stages of their biosynthesis enables the management of corresponding processes. This management can increase the content of not only Fcx itself but also other valuable pigments in diatoms. Therefore, biosynthetic studies of Fcx provided approaches which may increase the availability of **4** and allow the target application of metabolic engineering, for example, through overexpression of the pathway-specific enzymes and the enhancement of availability of their substrates [35]. As a good example, overexpression of VDL1 in the strain of *P. tricornutum*, which increased Fcx content, may be mentioned [29]. Another similar case concerns ZEP3 responsible for catalysis of diadinoxanthin conversion into diatoxanthin. Knock-out of the corresponding gene using CRISP/Cas9 technology led to the high light accumulation of diatoxanthin in the zep3 mutant strain of *P. tricornutum* [36].

The diatoxanthin was previously shown to be a potential therapeutic agent with antioxidant and anti-inflammatory properties for the treatment and prevention of severe inflammatory syndrome (so-called Cytokine storm) during SARS-CoV-2 infection [37]. Among several fucoxanthin-based products, the majority of food supplements, elaborated in different countries, FucoVital (Algatechnologies Ltd., Kibbutz Ketura, Israel) deserves a special mention as the first type of such preparation, which obtained approval from the USA Food and Drug Administration. FucoVital, derived from the diatom *P. tricornutum*, maintains liver health and can be used for obesity [38]. A series of recent reviews and experimental communications focused on research related to the pharmaceutical properties of Fcx including antibacterial, antioxidant, anti-inflammatory, skin-protective, anti-obesity, anti-diabetic, and other useful activities as well as its bioavailability and stability [39,40,41].

Marine carotenoids and particularly Fcx are well known as excellent antioxidants, capable of neutralizing the excess of free radicals and reactive species of oxygen and/or nitrogen. Elevated levels of the latter are characteristic of a wide range of metabolic, neurodegenerative, and other diseases. Fcx protects against oxidative damage more effectively in comparison with α-tocopherol. Its health benefits for the prevention of such diseases without adverse effects were shown [39,40,41]. It can be inferred that Fcx has undoubtedly been proven to be one of the most promising marine natural products, having biological properties of great interest. The diatoms themselves, particularly the *Phaeodactylum cruentum,* are the most promising candidates for superproducers of this valuable pigment.

#### 2.1.2. Marennine (Mar)

Biologically active pigment of another type, the so-called marennine, has been known for a long time as a metabolite of the widespread species complex of the pennate diatoms belonging to the genus *Haslea*. Diatoms from the genus *Haslea* can cause a global phenomenon of the “greening” in oysters, which is related to the effects of marennine. Such a phenomenon was observed in the Marennes-Olérgon region (Western France), where delicious “green oysters” feeding on the diatom *Haslea ostrearia* have been cultivated for centuries. The “greening” of oyster gills increases the market value of these mollusks [42]. Along with *H. ostrearia*, several new closely related species (*Haslea karadagensis*, *Haslea silbo*, *Haslea Nusantara*, and others), also containing similar pigments, have been recently described from various geographical areas of the World Ocean [43]. Different marennine-containing solutions, such as partially purified extracellular marennine (EM), concentrated supernatant (CS) from *H. ostrearia* culture, and the blue water (BW) were tested as potential antimicrobial agents followed by examination of their action on different marine invertebrates. It was proven that solutions such as CS and BW inhibit the growth of pathogenic bacteria *Vibrio chagasii*, *Vibrio crassostreae*, and other pathogens in aquacultures. They act as allelopathic agents on the early developmental stages, such as embryos and larvae, of different marine organisms, inhabiting areas used for the mollusks’ cultivation, whereas adults of invertebrates remain unaffected. However, the practical application of such biopreparations in aquaculture requires further examination [44].

There are two forms of Mar: intracellular (IMn) in diatom vesicles, and extracellular (EMn) in the culture medium. EMn shows diverse biological activities, including antioxidant properties and antibacterial and antiproliferative activities, with potential application in the food, feed, cosmetics, and health industries [45,46].

Structure studies of these types of pigments were in the focus of attention for a long time and, as a result, the polysaccharide nature of the skeleton of marennine pigments was proposed. However, the exact structure of this bioactive compound, particularly its structural fragment responsible for the green-blue color, has remained unknown. In structure analysis of this pigment, different glycoside hydrolases were used, but Mar was hydrolyzed predominantly by endo-1,3-β-glucanase. Application of nuclear magnetic resonance (NMR), mass spectrometry (MS), and a calorimetric assay showed that Mar might consist of a triple-helical complex of β-1,3-glucan molecules similar to those of the known laminarins from brown algae. Its rather low molecular weight (9.8–10 kDa) was determined by mass spectrometry. Some small signals at 7–8.5 ppm in the NMR spectra of marennine hydrolysates probably suggest the aromatic nature of a chromophoric group in this pigment. However, the exact chemical structures of this pigment and/or closely related compound were not determined [47,48,49].

After suggestion that Mar is a polysaccharide derivative, not only the effects of Mar but also the extracellular polysaccharides from *H. ostrearia* on dermal fibroblast skin cells and the expression levels of genes related to skin hydration, elasticity, and barrier function were studied, demonstrating their positive action on the skin barrier through improvement in hydration properties. The studied preparations demonstrated the promising potential in cosmetic formulations, being rich in ingredients and offering hydration, anti-aging, and antioxidant properties at low concentrations [50].

It is likely that the main problem of the structure determination of Mar is insufficient separation and purification of pigment preparations, even when they are obtained by large-scale culturing of producers with the improved solid-phase extraction [51]. This is the only case among the studies on diatom metabolites where a long-term project has not yet led to a solution of the problem of structure determination for either the target pigment or chromophoric group in the polysaccharide or oligosaccharide molecule.

### 2.2. Chrysolaminarins (Chrls)

**Scheme 2:**
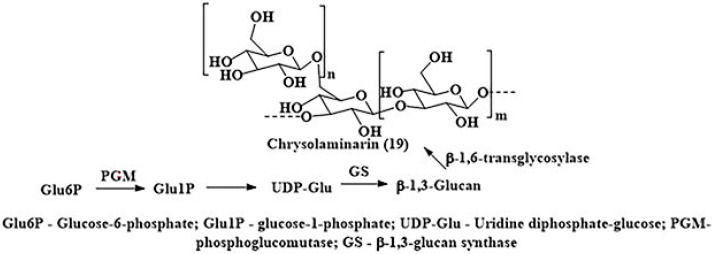
Biosynthesis and structure of chrysolaminarin (**19**) from the diatom *Phaeodactylum tricornutum*. Glu6P—Glucose-6-phosphate; Glu1P—glucose-1-phosphate; UDP-Glu—uridine diphosphate glucose; PGM—phosphoglucomutase; GS—β-1,3-glucan synthase.

The success of diatoms among phytoplankton microalgae was partly realized during their evolution by effective photosynthesis with the production of photosynthetically assimilated carbon in the form of polysaccharides. In contrast with higher plants, which synthesize and accumulate a reserve of high molecular α-1,4-glucans, diatoms produce low molecular bioactive β-1,3;1,6-glucans, so-called chrysolaminarins, as reserve polysaccharides. They are accumulated at photosynthesis, mobilized in the dark, and localized in a large cytoplasmically localized vacuole. Their content in diatoms is very high and may reach up to 60–70% of the dry weight of the microalgae [52]. Structurally, Chrls resemble laminarins from brown macrophytes, both constructed from a linear β-1,3-glucan backbone occasionally branched with additional glucose or oligoglucan units at the C-6 positions. The degrees of polymerization of these low molecular polymers are variable, depending on different species from 5 to 25 with an average of 20. To unravel the biosynthetic capabilities of diatoms, the comparison of diatom glucans with other related structures, particularly produced by the model diatom *P. tricornutum*, was carried out. Biosynthesis of Chrls (**19**) from uridine diphosphate glucose (UDP) includes two main stages: (1) synthesis of linear β-1,3-glucane, catalyzed by 1,3-β-glucan synthase (GS); and (2) synthesis of low molecular and water-soluble branched 1,3/1,6 glucan, catalyzed by 1,6-β-transglucosylases (TGS) (Scheme 2). Glucan synthases are two domain proteins with UDP glucose diphosphorylation and phosphoglucomutase (PGM) activities, which provide the activated glucose units and their polymerization [53]. Expression of the PGM domain catalyzes glucose phosphates interconversions that leads to the remarkable elevation in Chrls content in *P. tricornutum* and shares the carbon-rich precursors between carbohydrate and lipid metabolisms, reducing the lipid content and orchestrating Chrls and lipid biosynthetic pathways [54].

Antisense-based knockdown lines of GSs were generated in the diatom *Thalassiosira pseudonana*; at that, the knockdown of encoding genes decreased the Chrl biosynthesis, but transiently increased triacylglycerol (TAG) levels [55].

1,6-β-transglucosylases (TGS) are known as enzymes, catalyzing the 1,6-branching in different β-1,3 glucans, including Chrls. Two potential 1,6-β-transglycosylases (TGS) of the diatom *P. tricornutum* were found to be similar to yeast Kre6 proteins. Genetic fusion of these full-length TGS to the green fluorescent protein followed by expression of these constructions in *P. tricornutum* demonstrated that these enzymes are localized in vacuoles, and the stage of chrysolaminarin branching also occurs in these organelles [56]. Chrls biosynthesis in this diatom was enhanced by overexpression of 1,6-β-transglycosylases, confirming the crucial role of these enzymes in the biosynthesis of Chrls [53]. The content of Chrls in the same model species was increasing during the day and decreasing at night, with the similar fluctuation observed in neutral lipid levels, because the stored energy is spent for cell division during the dark period [57].

Diversity and structure peculiarities of Chrls depend on their producers and vary in different species of diatoms (Table 1). Chrls were shown to be attractive for aquaculture by their useful properties and biological activities. These compounds from the diatom *Odontella aurita* were isolated by DEAE-52 cellulose anion-exchange chromatography and Sephadex G-200 gel-filtration chromatography. The purified fraction, named CL2 with MW of 7.75 kDa, showed antioxidant properties [58]. These Chrls can be used as food constituents in the aquaculture of juvenile fish and other kinds of seafood. For example, the presence of the diatom *O. aurita*, accumulating Chrls, in the diets of juvenile golden pompano *Trachinotus ovatus* improved the growth, immune response, antioxidant capacity, and hepatic health of the fish. Really, the application of experimental food that contained 5% of *O. aurita* powder boosts the growth of these fish, increases the activities of several intestinal enzymes, promotes antioxidant capacity, and exerts hepatoprotective effects. As a result, such a diet was recommended to enhance the growth, antioxidant ability, and immunity of *T. ovatus* [59].

The corresponding biopolymer from *Conticribra weissflogii*, studied by NMR spectroscopy, differs from other Chrls by a low proportion of 6-substition by single β-glucose units. It enhances the phagocytic activity of macrophages and, therefore, possesses immunostimulatory activity [60]. Taxonomic distribution and structure peculiarities of Chrls in different diatoms are given in Table 1.

Some additional data concerning Chrls were discussed in the recent reviews of Chen et al. [61] and Kwasiborski et al. [62], who described Chrls biosynthesis and degradation for a better understanding of the possibilities of a more comprehensive use of these products. Considerable attention was paid to their use in the food, cosmetics, and pharmaceutical industries [61,62].

In our opinion, the structural diversity of Chrls and their immunostimulatory activity, especially in relation to higher-molecular-weight compounds, require further research. For example, it is unclear whether the molecular weights and degrees of branching in these compounds depend on the taxonomic position of their producers. At the same time, the basic principles of their biosynthesis are relatively well-understood.

### 2.3. Lipids

#### 2.3.1. Fatty Acids (FAs)

**Figure 2:**
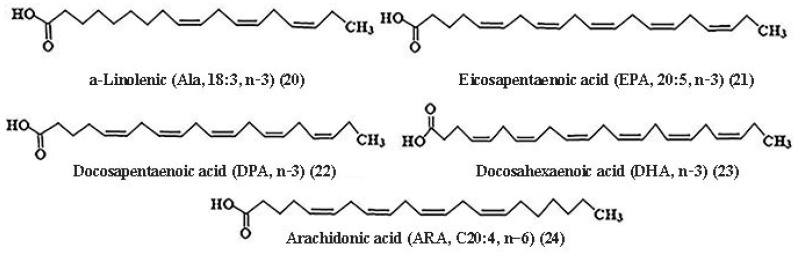
Some important PUFAs, belonging to (n-3) and (n-6) families.

Recently, it became clearer that fatty acids (FAs) are not only obligatory constituents of lipids, but also play other important biological roles [63]. The majority of FAs in diatoms differ from each other in the length of hydrocarbon chains and the number of double bonds in them, varying mainly from C12:0 to C22:6. The FAs, containing two or more double bonds and known as polyunsaturated ones (PUFAs), are widely represented in diatoms. In particular, PUFAs belonging to the omega-3 (n-3) and omega-6 (n-6)-families attract more attention due to their significance for human health. All-cis-9,12,15-octadecatrienoic acid (α-linolenic acid, ALA) (**20**) and the complex lipids, contained (**20**) are constituents of many edible products of terrestrial origin, for example, some seeds and oils. At the same time, **20** is also capable of transforming into marine lipids, and its consumption with food is required for human life. Eicosapentaenic C18:5 (EPA) (**21**), docosapentaenic (C20:5) (**22**), and docosahexaenic acids C20:6 (DHA) (**23**) are characteristic FAs of marine protists, diatoms, and some other microalgae, and are also essential for human health, being delivered with seafood. Arachidonic acid (**24**) belonging to the (n-6)-series is known as a biosynthetic precursor of important oxylipins in marine and terrestrial organisms (Figure 2). These long-chain polyunsaturated fatty acids (LC-PUFA) are capable of alleviating health conditions at arteriosclerosis, hypertension, inflammation, and microbial, viral, and tumor diseases.

Similar FAs having hydrocarbon chains longer than 24 carbon atoms are considered to be very long-chain unsaturated acids (VLC PUFAs). They attracted attention by the positive action on the integrity of the retinal membrane and prevention of macular degeneration and inherited retinal diseases [64].

The composition of lipids in diatoms varies in different culture media. Optimal conditions for obtaining good yields of biomass, total lipids, and increased levels of valuable FAs in two strains of the diatom *Skeletonema menzelii* have been found. In these cases, the accumulation of total lipids increased with a lack of N, P, and Si, but at a moderate amount of Fe. On the contrary, the highest content of PUFAs and EPA was achieved at a high content of N and P, a moderate amount of available iron, but a lack of Si in the media [65].

Diatom lipids are enriched with medium-chain FAs as well as with very long chain-polyunsaturated fatty acids (VLC-PUFAs) such as EPA (**21**). Given that C16 FAs can account for nearly 100% of the total FAs at the sn-2 position of triacylglycerides, the latter are promising for both biodiesel production and the preparation of biologically active compounds [66,67].

**Scheme 3:**
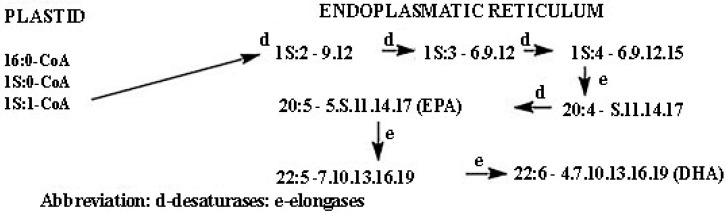
Long-chain (n-3) fatty acid biosynthesis pathways in the model diatom *Phaeodactylum tricornutum* and compartmentation of the biosynthesis [68], d-desaturases; e-elongases.

Isolation of c-DNA of some genes of FAs biosynthesis followed by their overexpression allowed increasing the content of lipids, produced by diatoms. The discussion on pathways and key catalyzing enzymes of FAs biosynthesis concerned the processes proceeding in the chloroplasts (so-called prokaryotic pathway) and in the endoplasmatic reticulum of diatoms (eukaryotic pathway). FAs eukaryotic biosynthesis starts from the desaturation of plastidic 18:1FA, converted into 18:2(n-6) FA. A series of desaturation and elongation stages take a place in the endoplasmatic reticulum, at catalysis by different desaturases and elongases, which leads to the formation of a diverse set of FAs, including mid chains (14–16 carbons) and long chains (18–22 carbons) such as those in (Scheme 3) [67]. FAs with longer chains (24 carbons or more) differ from others in resistance to oxidation and other properties.

The most valuable LC PUFAs in higher marine animals originate from marine microalgae and protists. These PUFAs, which enter the animals via the food chain, are subsequently found in seafood, and ultimately reach humans through processed products such as fish oil. Fractions, containing (n-3)-PUFAs, can be isolated from diatoms by extraction techniques. For example, cultures of *Pseudostaurosira trainorii* were submitted to Soxhlet extraction using methanol–chloroform, reflux extraction with acetone, and supercritical fluid extraction (SFE) using 96% ethanol. Although the yields of EPA and DHA obtained as a result of SFE application were lower, their toxicity was absent and the obtained fractions could be used in dietary compositions without additional treatment [68].

Recently, (n-3) PUFAs, including EPA (**21**) and DHA (**23**), were studied on 527 patients at the doses of 2 g/day and purity ≥ 90% to reveal cardiometabolic risk factors related to the clinical applications. At that, **23** improved vascular function better than **21** and reliably lowered heart rate and blood pressure [69].

The n-3 docosapentaenoic acid (DPA) (**22**) is less studied in comparison with **21** and **23** because it usually presents in marine biological sources in smaller quantities. However, the **22** has always been a part of healthy nutrition for infants, who obtain these useful FAs from human milk. Grass-fed beef also contains the n-3 docosapentaenoic acid. Moreover, **22** and **23** are precursors of a large series of hydroxylated lipid mediators (protectins, resolvins, maresins, isoprostanes), which are implicated in the pro-resolution of the inflammation. In addition, **22** is the most abundant PUFA in the human brain after **23** and is active in elderly neuroprotection and early-life development. The (n-3) DPA may be considered as a storage depot for **22** and **23** in humans. These, and other findings and useful properties, resulted in the growing interest and even the practical application of these FAs in humans in some food supplements [70,71].

Different approaches were elaborated to solve the problem of the supply of **22,** including its isolation from new natural sources and synthesis. Moreover, it was discovered that heterokont protist *Schizochytrium* sp., belonging to the family Thraustochytriaceae, produces significant amounts of DPA (**22**) and particularly DHA (**23**), providing opportunities to accumulate these FAs for in vivo investigation. *Schizochytrium* sp. proved to be the best microorganism for the industrial production of **23** due to easy cultivation, fast growth rate, and high content (about 40% of total FAs). *Schizochytrium* sp. has attracted increasing attention in the field of biotechnology due to its diverse potential applications as a source not only (n-3) LC PUFAs, but also as a promising candidate for obtaining valuable carotenoids and polysaccharides [72,73]. DPA (**22**) and DHA (**23**) are also promising as drugs against neuroses. For example, (n-3) DPA supplementation in rats in the dose of 150 mg/kg/day for 6 days leads to a reduction in symptoms associated with depression and may improve neural health [74].

Intact diatom *P. tricornutum* does not accumulate elevated levels of **23**, but the transgenic Pt_Elo5 strain, genetically engineered by overexpressing heterologous genes, encoding enzymes in the LC-PUFA biosynthetic pathway, was more productive. Production of LC-PUFAs increases in a raceway pond, supplemented with artificial illumination on a 16:8 h light/dark cycle, in natural seawater with F/2 nutrients led to 24.8% EPA (**21**) and 10.3% DHA (**23**). Therefore, this biological source is also quite suitable to accumulate lipids enriched with valuable PUFAs [75]. Another variant of genetically engineering *P. tricornutum* with the inactivation of hotdog fold thioesterase gene produces 1.7-fold more TAG than wild strains, and contains mainly short FAs in TAG suitable as biodiesel [76]. On the basis of experiments with the 13C-isotope labeling, it was suggested that the diatom *Chaetoceros muelleri* produces **21** by a combination of the n-3 (via 18:4n-3) and n-6 (via 18:3n-6 and 20:4n-6) biosynthetic pathways as well as by the alternative ω-3 desaturase pathway (via arachidonic acid 20:4n-6 FA [77]).

Probably, the most interesting information about the FAs of diatoms mainly concerns the pharmaceutical action of some of them.

#### 2.3.2. Triacylglycerols (TAGs)

In the last decade, at least two main scientific directions related to neutral lipids, primarily to TAGs from diatoms, have been developed in depth by the following: (i) obtaining new data concerning the diversity, biosynthesis, and metabolism of this type of neutral lipids using modern molecular genetics methods, and (ii) via examination of peculiarities of the storage of these metabolites in lipid droplets and the subsequent fate of these oil bodies, which have some characteristics specific to diatoms. Over the years, oil-rich diatom species, which contain significant amounts of TAGs have received an attract attention due to their ability to accumulate these key components with good potential for the use as biofuels. Attempts to completely separate triglyceride sums from diatoms with the isolation of individual compounds have not yet been successful, although the studies of mixtures of neutral lipids have yielded interesting results. The predominant FAs in neutral lipids of diatoms were identified as 14:0, 16:0, 16:1, and 20:5 with C18 fatty acids, present only in trace amounts. It is good for biofuel production, because lipids with major medium-chain FAs result in less viscous biodiesel [66].

To boost TAG levels, which is important for biofuel production, several strategies were applied. Nitrogen and iron deficiency as well as silica depletion redirect carbon flux toward biosynthesis of TAGs as storage lipids. Lipid accumulation may also be realized as a result of blocking the competing pathway of Chrls biosynthesis. Finally, knockdown of lipid catabolism is one more approach influencing TAG production [78].

**Scheme 4:**
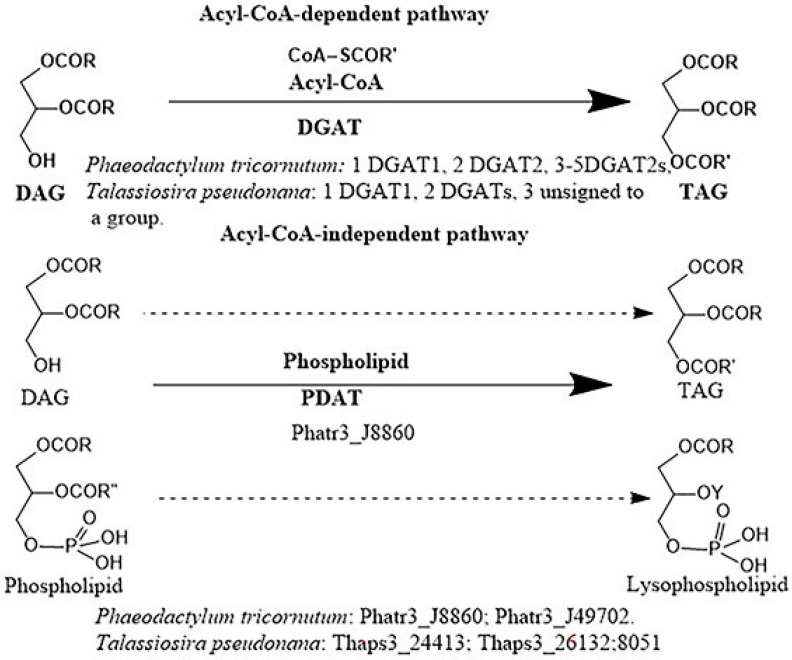
Triacylglycerols biosynthesis pathways in the diatoms *Phaeodactylum tricornutum* and *Thalassiosira pseudonana* (based on data, reported in [81]). DAG—dyacylglycerol, TAG—triacylglycerol, DGATs—diglycosylacyltransferases, which catalyze the transfer of one chain from acyl-CoA to TAGs; PDAT—phospholipid/diacylglycerol acyltransferases, which transfer one chain from a phospholipid to triacylglycerols.

Using a genomic approach, novel information on TAGs biosynthesis and metabolism was primarily derived from studies on three model species: *P. tricornutum*, *T. pseudonana*, and *Fistulifera solaris*, which contain lipids constituting 30%, 31%, and 60% of their total biomass, respectively. *P. tricornutum* is the only diatom species studied at the ecotype levels with 17 established ecotypes from Pt1 to Pt17 [79,80]. The majority of molecular genetic studies were conducted on Pt1 ecotypes. Biosynthesis of TAGs in diatoms proceeds from diacylglycerol (DAG) by two known pathways: i) the Acyl-CoA-dependent pathway; and ii) the Acyl-CoA-independent pathway, catalyzed by acyltransferases (DGATs) and phospholipids/diacylglycerol acyltransferases (PDATs), respectively (Scheme 4). Seven DGATs were found in *P. tricornutum*, while six DGATS encode TAG formation in the genome of *T. pseudonana* [81].

Recently, Zhang et al. [82] performed heterologous expression of DGAT2 in yeast, followed by the increased expression of this enzyme in *P. tricornutum*. They have demonstrated that the PtDGAT1 strain outperforms other DGATs in TAG synthesis. Compared to the wild-type of *P. tricornutum*, the PtDGAT1-overexpressing strain produced more than twice as much TAG and total lipids, namely 57% and 73% of the dry weight, which are the highest levels ever achieved in this species.

TAG synthesis occurs in both plastids (first stages) and the endoplasmatic reticulum (ER) by the above-mentioned Kennedy pathway with intermediate diacylglycerols. TAGs are synthesized in lower plants from glycerol-3-phosphate (G3P) and diacylglycerol (DAG). A third acyl chain is introduced in TAGs mainly in the ER, the first stages of this synthesis are common to all glycerolipids and can occur both in plastids and the ER. TAGs are hydrophobic water-insoluble neutral lipids, which must be separated from other cellular constituents. They are the main forms of neutral lipids in diatoms, which are present within spherical oil drops, also called lipid-storage droplets (LDs). Maeda et al. [83] described the number, size, and morphology of oil bodies in diatoms, organized as characteristic spherical organelles with the TAGs, phospholipids, lipids, and proteins they contain. The number of TAG-containing LDs varies in different diatom species from 1–2 to 10–15 depending on species, environmental conditions, and the growth phase of a diatom. These organelles bear not only TAGs, which are accumulated, particularly when a diatom is exposed to nutrient deprivation conditions, but also other insoluble compounds, for example, carotenoids, including Fcx, β-carotene, and diadinoxanthin. Lipid droplets are surrounded by single-layer membranes, constructed mainly from phospholipids, glycolipids, sterols, phosphatidylcholine, and some proteins such as the Stramenopile-type lipid droplet protein [84,85]. Lipid droplets are presented in the majority of living organisms. The degradation of LDs proceeds using lipolysis and lipophagy. Lipolysis occurs at the surface of LDs, releasing FAs bonded to the glycerol in neutral lipids. Lipophagy is known as the degradation of LDs by autophagy in autophagosomes. In eukaryotic cells, the LDs’ degradation is a key process for release of TAGs and FAs. The diversity, complexity, and specific degradation mechanisms of LDs were only recently elucidated [86].

For diatoms, extensive investigation of LDs was implemented mostly on *P. tricornutum*, and to a lesser extent on *T. pseudonana*, including LD formation and degradation mechanisms. In their recent review on diatom TAG biogenesis, metabolism, and accumulation, Murison et al. [81] concluded that in-depth research in this scientific field became possible only in the last two decades with the help of “-omics” approaches and genetic engineering technologies. In contrast to oil bodies of higher plants, microalgal LDs function as not long-term material and energy stores, but rather as transient reservoirs, where storage lipids are degraded in quick response to environmental changes [78].

TAGs are one of the structural groups of diatom lipids that have attracted the most attention over the past decade. However, so far, they have mainly been found in marine species, while freshwater diatoms are less studied in this respect.

#### 2.3.3. Glycolipids (GLs)

Microalgae are rich sources of glycerolglycolipids (GLs), prospective for various applications in medicine, cosmetics, and in food compositions. In diatoms, GLs like those from other plants comprise natural products such as monogalactosyldiacyl glycerolipids (MGDGs, **25**), digalactosyl diacylglycerols (DGDGs, **26**), and sulfoquinovosyl diacylglycerolipids (SQDGs, **27**), with their different molecular forms, content, and relations of which directly depend on the taxonomic status of producers and growth conditions. Diatom GLs are rich in 16- and 18-carbon saturated and unsaturated fatty acids and, as a rule, also contain PUFAs like n-3 α-linolenic (**20**), docosahexaenoic (**23**), and particularly eicosapentaenoic (**21**) acids. Some lipids of this class show antitumor, antimicrobial, and anti-inflammatory activities and also have important nutritional significance. Lipidomics studies demonstrated GLs to be an exclusive reservoir of various glycolipids [87]. Considering the huge chemical diversity of GLs and easy cultivation of some of their microalgal producers, it was suggested that diatoms could also be new promising sources of immunomodulatory molecules [88].

**Figure 3:**
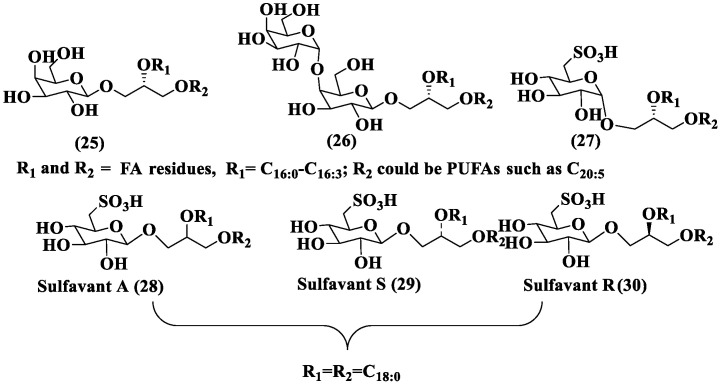
Some glycolipids from diatoms and their synthetic analogs.

A new immunomodulatory sulfolipid, a synthetic analog of natural SQDGs called β-SQDG18 or Sulfavant A (**28**) (Figure 3), can activate human dendritic cells (hDCs) that recognize foreign substances, stimulating the adaptive immune system. This compound acts via a TLR2/TLR4-independent mechanism and triggers immune response in vivo. Immunization of mice against ovalbumin, using β-SQDG18 as an adjuvant, produced an increased titer of anti-OVA Ig, like traditional adjuvants. In an experimental model of melanoma, analogous immunization of C57BL/6 mice elicited a protective response with a decrease in tumor growth and an increase in the animal survival [89].

To obtain the Sulfavant A (**28**), an analog of the glycolipid with general formula (**27**), as a lead compound in sufficient amount for preclinical development, the modified and improved chemical synthesis of **28** was carried out. The procedure included 11 steps and led to 17% overall yield of the target compound on initial D-glucopyranose (two-fold higher than in preliminary synthesis), and it also allowed the obtaining other analogs, sulfavants S (**29**) and R (**30**) [90]. The fast and sensitive method, for the measurement of Sulfavant A content, based on UHPLC-HRMS, was developed and validated over a range of concentrations 2.5–2000 ng/mL with d70-derivative of **28** as internal standard. The chromatographic column UHPLC Kinetex^®^ (Phenomenex, Torrance, CA, USA) 2.6 µm PS C18 column and a gradient of methanol in 0.32 mM ammonium hydroxide solution (pH 8) as well as mass spectrometry with electrospray ionization (ESI) (negative mode) were used. The lowest limit of determination of Sulfavant A was achieved at 6.5 ng/mL [91].

To establish the mechanism of Sulfatant A action, the determination of its molecular target in human dendritic cells (hDCs) was necessary. Using 2B4 GFP-NFAT reporter T cells stably transfected with human TREM2, SULF A was shown to bind to a triggering receptor expressed on myeloid cells 2 (TREM-2) and induce selective TREM2 signaling. SULF A, as an agonist of TREM 2, initiated an unconventional maturation of hDCs followed by enhancing the migration activity and upregulation of major histocompatibility complex (MHC) with hypoproduction of cytokines. Binding to TREM2 caused the differentiation of hDC into a novel homeostasis-determining phenotype (homeDCs). Its action was compromised by the blockade of TREM-2 at its interaction with goat anti-human TREM2 antibody and silencing of the TREM2 gene through interference with siRNA in hMoDCs. Activation by SULF A preserved the hDCs functions to excite the allogeneic T cell response and increased interleukin-10 release after lipopolysaccharide stimulation. Therefore, TREM-2 proved to be the molecular target of the adjuvant action of SULF A [92]. The molecule was proposed as a vaccine adjuvant (EU Patent n. EP3007725B1).

The immunoregulatory effect of Sulfatant A was further investigated in human allogenic mixed lymphocyte reaction (MLR). This reaction between human monocyte-derived dendritic cells and naïve T lymphocytes, derived from six different donors, was used as a test to assess the immunoactivity of the lead compound. Supplementation of 10 µg/mL SULF A to the co-cultures induced expression of the costimulatory molecules ICOSL and OX40L and reduced release of the pro-inflammatory cytokine IL-12. After 7 days of SULF A treatment, T lymphocytes were more actively proliferating, which increased IL-4 synthesis and downregulated Th1 signals such as IFNγ. Consistent with these data, naïve T cells were polarized into a regulatory CD4+ phenotype with upregulation of FOXP3 expression and IL-10 synthesis. Thus, it was shown that SULF A can modulate DC-T cell synapse and promote a regulatory response in the MLR test. The molecule triggers a negative modulation of the immune synapse by expression of co-inhibitory molecules on DCs and T cells, along with the release of IL-4 and IL-10 cytokines, promoting the weakening of inflammation [93].

Stereoisomeric Sulfavants A and R are under study as vaccine adjuvants. Their in vitro adjuvant activities were shown to be quite different, since Sulfavant A gave the best activity at about 20 µM, Sulfavant R at 10 nM. A probable explanation of this difference consists of their self-assemblies in aqueous systems. At the nanomolar range, Sulfavant A formed cohesive vesicles, while Sulfavant R presented as spherical micellar particles whose reduced stability was probably responsible for an increase in its monomer concentration [94]. Generally, the studies on sulfavants are probably the most interesting examples of the research on GLs from diatoms in the last decade.

#### 2.3.4. Phosphoglycolipids

**Figure 4:**
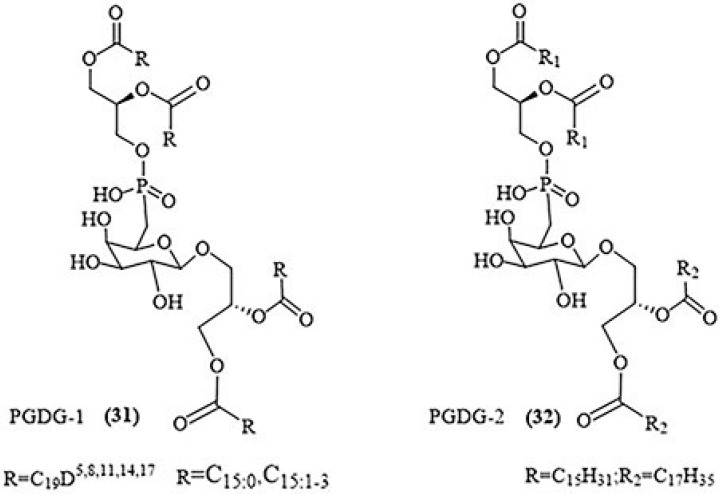
Phosphatidylmonogalactosyldiacylglycerols.

In 2019, unprecedented phosphatidylmonogalactosyldiacylglycerol (PGDG-1, **31**) in which two glycerol residues, acylated by FAs are attached to a phosphate group of D-galactopyranose-6-phosphate and to C-1 of the same monosaccharide by β-glycosidic bond, was isolated from the methanol extract of the diatom *T. weissflogii* by bioassay-guided LH-20 gel chromatography followed by HPLC. This natural product demonstrates immunostimulatory action in human dendritic cells, advancing the maturation of DCs and enhancing the expression of IL-6, IL-8, and IL-12p40 interleukins at concentrations of 5 mg/mL or higher. To examine peculiarities of its immunostimulatory activity, a new model PGDG-2 (**32**) was synthesized by a 15-step synthesis from commercial D-galactose (Figure 4). The obtained compound was proven to be an agonist of toll-like receptor-4 in human and murine DCs and demonstrated antigen-specific T cell activations [95].

Later, the same Italian group described a new approach for the search for new natural products with anticancer immunotherapeutic activities [96]. These works attracted attention and were cited in at least four reviews in the last 3 years [97,98,99,100].

#### 2.3.5. Oxylipins (OLs)

Compounds that belong to this structural group, like a number of other diatom metabolites, can be considered as endometabolites in diatoms themselves and simultaneously as exometabolites in consumers, which they transferred via food chains. OLs belong to a separate class of lipids and were studied in both macroalgae and microalgae, including diatoms. They also present in higher plants and animals. As chemical defense agents, some OLs in diatoms are rapidly produced after mechanical disruption of their cells, which can occur as a result of grazing or infection. For some time, lypoxygenases (LOXs) of different specificity keep their activity also in seawater, converting PUFAs to hydroperoxides and other products such as unsaturated aldehydes and hydroxylated FAs, for example, hydroxyeicosapentaenoic acids (HEPEs). The majority of OLs in diatoms are derived from the C16, C20, C22, and to a lesser extent from C18 PUFAs followed by conversion of the resulting hydroperoxides into the above-mentioned and other secondary products that play important and mainly defensive biological roles [101].

**Scheme 5:**
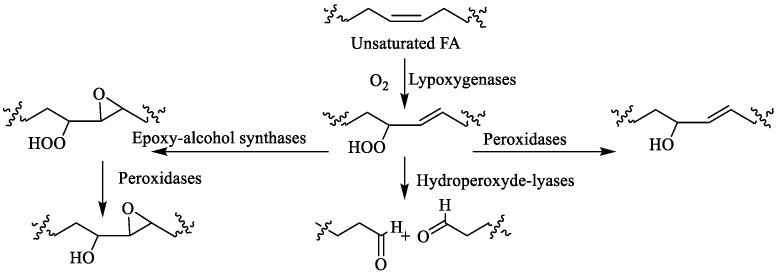
Some enzymatic transformations of unsaturated FAs, initiated by the action of LOXs.

OLs of diatoms are natural defensive agents, generated by the introduction of oxygen atoms into PUFAs at the catalysis by LOXs, with subsequent conversions catalyzed by a variety of other enzymes such as hydroperoxide lyases (HPLs), peroxidases, reductases, etc.; they are consequently converted into alcohols, epoxidases, aldehydes, and other derivatives (Scheme 5).

There are two major structural groups of these physiologically and ecologically active natural products: (i) non-volatile oxylipins, preserving the long chains of their precursors, and (ii) so-called volatile OLs such as polyunsaturated aldehydes (PUAs) with shortened chains.

**Figure 5:**
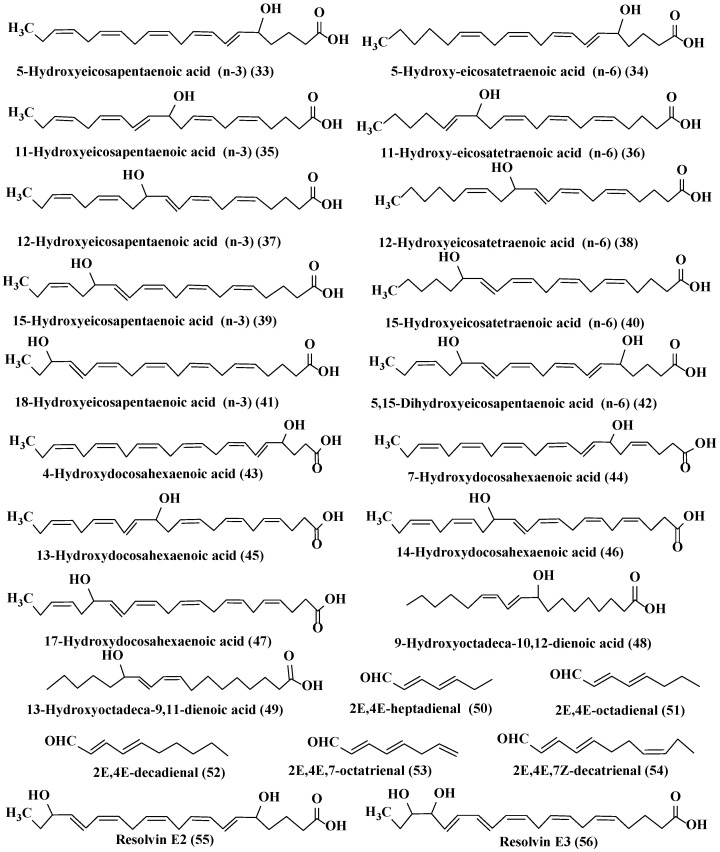
Non-volatile oxylipins (**33**–**49**), derived from eicosapentaenic, arachidonic, and docosahexaenic acids, and PUAs (**50**–**54**) as well as resolvins (**55**, **56**) from the diatoms *Coscinodiscus granii*, *Thalassiosira rotula*, *Skeletonema costatum*, and *Chaetoceros didymus* [102].

Recently, very complicated mixtures of non-volatile OLs were structurally identified in the diatoms *T. rotula* and *S. costatum*, previously known by their toxic PUAs, as well as in bloom-forming diatoms *Chaetoceros didymus* and *Coscinodiscus granii*. These diatoms were harvested upon the stationary phase of growth, centrifuged, and extracted on conditioned SPE columns. Elution was performed by methyl formate, then the target compounds were extracted by MeOH/H_2_O and analyzed by UPLC-MS analysis. For the detection of oxylipins, a QT trap MS instrument, operated with electrospray ionization in the negative mode, was used. Analytical data obtained by LC–MS/MS measurements indicated a surprising variety of oxylipins, including mono- and dihydroxylated C18-, C20-, and C22-PUFAs in these diatoms. Non-volatile OLs (**33**–**49**) were identified along with volatile PUAs (**50**–**54**), as well as the resolvins E2 (**55**) and E3 (**56**), earlier known in some mammalians cell signaling agents, orchestrating the resolution of inflammation in higher animals, which were found for the first time in non-mammalian biological sources (Figure 5). For OLs, which were not identified by comparison with standards, characteristic MS/MS fragment peaks, as well as extrapolation of the literature RT data, were used. The majority of identified compounds were presented by OLs, derived from EPA (**21**) and DHA (**23**) [102].

PUAs in diatoms show deleterious effects on herbivores, inhibit competing phytoplankton species, and increase rates of remineralization of bacterial organic matter. Their compositions depend on the trophic status of producers: decadienal (**50**) predominated in oligotrophic waters, octadienal (**51**) in eutrophic, and heptadienal (**52**) in intermediate conditions. The production of PUAs released by phytoplankton increased under oligotrophic conditions and in waters where a high ratio of dissolved inorganic nitrogen to dissolved inorganic phosphorus was observed [103].

Multiple biological roles of OLs were recently discussed in the review of Ruocco et al. [101]. Both non-volatile long-chain and volatile OLs play important roles in the chemical defense of their producers, being allelopathic and antibacterial agents, and participate in plant–plant and plant–animals cell-to-cell signaling in the environment. Paradoxically, diatoms, eaten by their consumers, for instance, herbivorous copepods, can simultaneously support copepod larval growth and reduce their fecundity and hatching success because of Ols’ toxic action. As a result of participation of the OLs in interspecies relations, they affect not only phytoplankton species, but also marine invertebrates feeding on diatoms. These ecologically active products also inhibit different organisms, competing with phytoplankton. Diatom blooms may induce a negative influence by their toxins on target consumers and competitors. Primary LOX products produce reactive oxygen species (ROS), inducing DNA and protein damage that contribute to cell aging and apoptosis. Response of copepods, sea urchins, and tunicates (main consumers of these microalgae) to diatom OLs was also studied on the genetic level. The upregulation of genes encoding enzymes involved in apoptosis was indicated mainly in larval stages of sea urchins (caspase8, caspases 3/7, NF-κB), induced stress of copepods (upregulation of HSP 70, CAT, and some other genes), and caused the development aberrations of tunicates (upregulation of gst and Aldh3,2,8 genes). It is noteworthy that some OLs exhibit anticancer, antimicrobial, and antituberculosis properties, while volatile OLs also show antiparasitic effects, opening up the prospects for their use [101].

It was again confirmed that bacteria–diatom interactions are very important for marine life. Actually, algicidal bacteria can lyse diatom blooms at the terminal stages of their development. For example, it was established that *Chaetoceros didymus* releases the eicosanoid oxylipins and shows lytic action against the algicidal bacterium (*Kordia algicida*)*,* inducing the production of wound-activated oxylipins in this resistant diatom. The prevailing hydroxylated fatty acid, 15-HEPE (**39**), was proven to be a potent inhibitor of the growth of *K. algicida* (inhibiting concentration is approximately 1 µM) [104].

Generally, signaling molecules, represented by various OLs, are the well-known chemical mediators in diatoms. For example, polyunsaturated aldehydes from the diatom *S. marinoi* demonstrate teratogenic activity, inducing apoptosis in the copepod *Calanus helgolandicus* feeding on this diatom. The decadienal (**52**) inhibits the growth of the centric diatom *T. weissflogii* and influences the abundance and species distribution of picoplanktonic communities in the surrounding environments [105].

**Figure 6:**
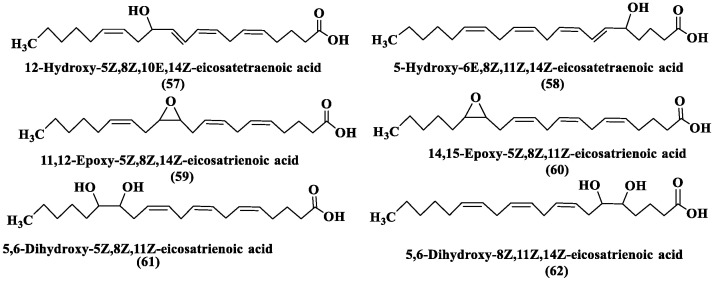
Some OLs, identified in the copepod *Calanus helgolandicus* in the end of the bloom of *Skeletonema marinoi*, April 2011, the North Adriatic Sea.

OLs, as signaling molecules, are released by microalgae in higher quantities into seawaters during diatom blooms, for example, it was at the end of a mono-specific bloom caused by *S. marinoi* in April 2011 in the North Adriatic Sea [106]. These biomolecules targeted zooplanktonic copepods, including the *C. helgolandicus.* An oceanographic cruise onboard the R/V Urania was used for the investigation of diatom/copepod interactions. A big series of OLs, including some new noticeable constituents **57**–**62,** were identified by LC/MS/MS analysis in phytoplanktonic samples (Figure 6). A series of possible defense mechanisms against the deterrent action of these compounds from diatoms has been identified. In particular, the reduction in survival, inhibition of egg production, as well as suppression of the hatching success with downregulation of genes involved in mitotic spindle formation, inhibition of cell division, and increased apoptosis were indicated in the copepod *C. helgolandicus* feeding on diatoms [106].

Long-chain OLs were recently renamed from “non-volatile lipids” to “the linear oxygenated fatty acids (LOFAs)” and analyzed by Italian teams, using the corresponding surface water phytoplankton samples, collected weekly for 1 year (2017) at the Long-term Ecological-Research Station Mare Chiara (40°48.5′ N, 14°15′ E). The approach, elaborated by the authors, obtained results, and their conceptual model would help to decipher the importance of allelochemicals such as OLs for the dynamics of phytoplankton in marine biological systems [107].

Diatom-derived OLs are characterized by a high structural diversity and a wide range of biological functions, which they exhibit primarily as infochemicals.

### 2.4. Prostaglandins (PGs)

Prostaglandins, belonging to the class of eicosanoids, are derived from PUFAs of membrane phospholipids at the action of phospholipases such as PLA2. They are produced not in specialized glands like genuine hormones in mammals, but in cellular membranes of many tissues and/or organs of the corresponding biological objects. In mammals, they are synthesized mainly from arachidonic acid (**24**, Figure 2) through the enzymatic pathway, initiated by cyclooxygenases (COXs), which belong to the heme-peroxidase protein superfamily. COXs exist in two isoforms: i) COX-1 from ER, which is constitutively expressed in many tissues, and ii) COX-2 as an inducible form, located in the nuclear envelope, which appears in response to different incentives such as inflammation, carcinogenic, and/or oncogenic factors. PGs provide not only anti-inflammatory responses, but also control many physiological and pathologic processes, for example, muscle and blood vessel tone, immune status, apoptosis, inflammation, and proliferation. These natural compounds, having hormone-like actions, were also found in a wide variety of marine organisms, including marine invertebrates such as Mollusca, Porifera, Echinodermata, Coelenterata, and Crustaceae, as well as in red and brown algae, functioning in all of them as mediators and defense agents [108].

**Scheme 6:**
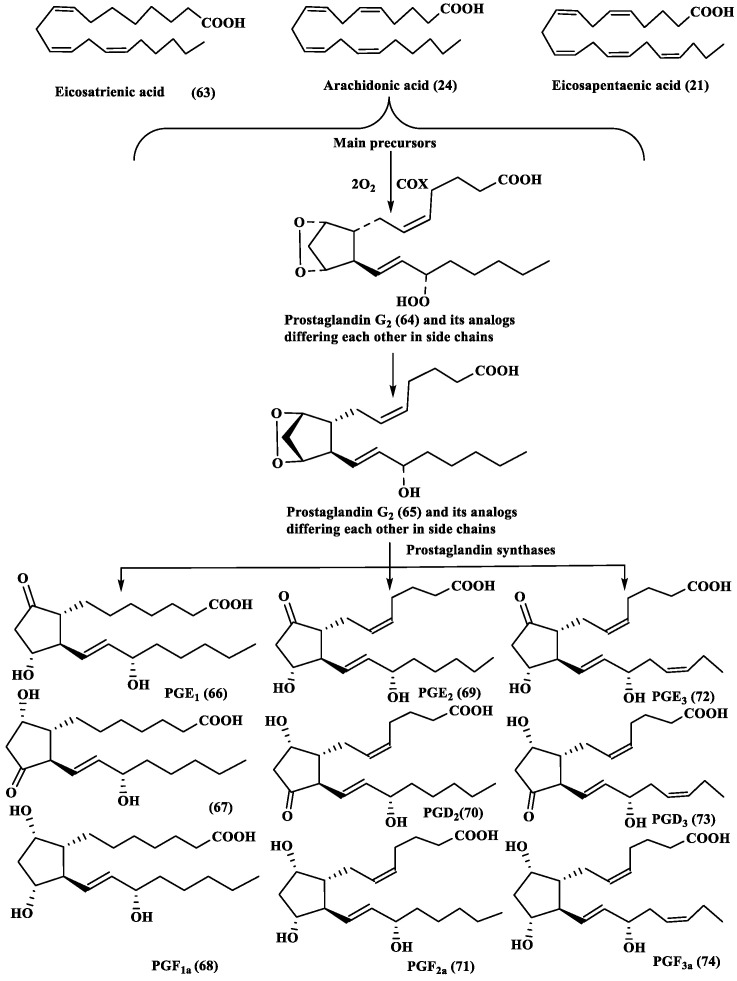
PG biosynthesis in diatoms, adopted from [109].

Recently, PG was found in diatoms for the first time. Really, as a result of these studies on transcriptomes of two strains (FE7 and FE60) of the diatom *S. marinoi*, an active prostaglandin biosynthetic pathway was established, being differentially expressed in both strains. The sequences of three main enzymes of PG biosynthesis, namely prostaglandin-endoperoxide G/H synthase 1 or cyclooxygenase-1 (COX-1), microsomal prostaglandin E synthase 1, and prostaglandin-H2 D-isomerase, along with the protein prostaglandin transporter, were established in the FE7 strain, while the COX-1 gene was not immediately determined in the FE60 strain. However, real-time-qPCR experiments confirmed the expression of all the transcripts in both strains. Moreover, the presence of various prostaglandin metabolites such as PgE3 and PGD3, found in *S. marinoi*, suggests that PGs of diatoms are biosynthesized not only from arachidonic (eicosatetraenic) acid (**24**), but also from EPA (**21**) as a main PUFA of diatoms. One more PUFA, namely eicosatrienic acid (**63**), may also be a precursor of some PGs in diatoms. Action of COX on the abovementioned PUFAs first leads to their rapid conversion through cyclization and inclusion of two oxygen molecules into the unstable PGG2 (**64**), and is subsequently quickly reduced to PGH2 (**65**). Further transformations of PGH2, catalyzed by prostaglandin synthases, yield three series of PGs, biosynthesized from eicosatrienic acid (**63**), arachidonic acid (**24**), and EPA (**21**). The first series includes such PGs as **66**–**68** and related compounds, the second consists of **69**–**71** and many other PGs, and finally, the third consists of **72**–**74**. A prominent contribution of the last series was detected in diatoms (Scheme 6). It was confirmed by LC/MS/MS that the FE7 strain was more productive by PGs than FE60. The discovery of a canonical animal pathway leading to prostaglandins in marine diatoms opened a possibility to trace the evolution of this important cell-signaling pathway from microorganisms to humans [109]. PGs in diatoms are probably involved in the prey–predator interaction because the prostaglandin pathway is activated in the diatom *S. marinoi* under grazer pressure [110].

In addition, transcriptome analysis allowed indicating of the prostaglandin biosynthesis by identification of PG G/H synthase, PGE synthase 2, and PGF synthase genes in the diatom *T. rotula*. The syntheses of other bioactive metabolites, such as polyketides and secologanin, also take place in this species, collected from the Gulf of Naples [111]. Later, the PG biosynthesis in the *T. rotula* was examined in detail, and differences in its transcriptome in comparison with those of the diatom *S. marinoi* were found. While the expression of key enzymes such as COX-1 and microsomal prostaglandin E synthase 1 was indicated in both species, the expression of prostaglandin H2 D-isomerase was proven to be only in *S. marinoi*. It was shown that enzymes responsible for PGs’ synthesis, such as COX prostaglandin E synthase 2 and prostaglandin F synthase, are mainly expressed at the end of the exponential phase, when PG biosynthesis is released mainly during the stationary and senescent phases, suggesting a possible signaling function for diatom PGs.

Phylogenetic analysis of the key enzymes, namely COX, indicated the presence of more than one copy of this enzyme, related to the oxidative metabolism of FAs and belonging to the peroxidase-cyclooxygenase superfamily in diatoms. Moreover, the release of PGs in the surrounding environment during different growth phases of the diatom *T. rotula* was assessed for the first time. These findings suggest a more complex evolution and a great diversity of metabolic pathways associated with this class of lipid mediators in diatoms [112].

Prostaglandin biosynthesis and metabolism in phytoplankton microalgae were recently reviewed. Some planktonic microalgae, including diatoms, could serve as a biotechnological platform to obtain PG-containing preparations for the treatment of chronic inflammatory diseases and in other clinical conditions. State-of-the-art clinical studies on the action PGs from eukaryotic marine phytoplankton were also discussed. Gene-editing techniques were applied to diatoms using transcription activator-like effector nucleases (TALEN), either the CRISPR method with a Cas9 nuclease, or by action of ribonucleoprotein complexes. It was noted that, at present, two centric diatoms, *S. marinoi* and *T. rotula*, are successfully used as model organisms for studies on PG biosynthesis. The diatom *P. tricornutum* contains not only PGs, but also isoprostanoids, which are produced via isomerization of PUFA precursors [113]. Studies on these species revealed the existence of a canonical animal PG pathway with specific peculiarities connecting with the participation of particular PUFAs such as EPA (**21**) and DHA (**23**) in their biosynthesis, and allowed the suggesting of their biosynthesis as a defense response to grazers [109].

Thus, in the last decade, PGs were first found and identified in diatoms, leading to new insights into the origin and evolution of this important group of natural bioregulators.

## 3. Exometabolites

Many diatom metabolites perform important ecological functions by transmitting chemical signals to other individuals of the same species or other species. They can be classified as ecologically active substances.

### 3.1. Sex Pheromones of Diatoms

The discovery of structures and functions of diatom sex pheromones is an outstanding finding important for all who study and are interested in these microalgae. It is well known that diatoms reproduce themselves using one of two possible procedures occurring in aqueous environments: either via asexual (by mitosis) or sexual reproduction (by meiosis). After multiple cycles of mitosis, the mean size of diatom cells becomes significantly smaller than their original size, and further mitosis is blocked or, if it continues, the cells die.

The diatom *Seminavis robusta* was selected as a model species to study sexuality in diatoms. The sexual stage of reproduction begins when the cells reach a suitable size below the so-called sexual size threshold (SST) (51.6 ± 0.5 μm). When the diatom passed SST, two sexual mating types appeared: migrating type (Mt^+^) and attracting one (Mt^−^), which can be distinguished by their behavior using light microcopy. Each of these cell lines produces sex-induced pheromones (SIPs), activating mating behavior in their partners. Moreover, Mt^−^ cells induce attractive pheromone (**75**), while Mt^+^ cells express receptors to this pheromone. As a result, MT^+^ cells become motile and attracted to a diproline source. This pheromone **75,** acting on the second stage of sexual reproduction, was found in the culture medium of Mt^−^ cells by comparative metabolomics, using a solid-phase extraction by a special hydrophilic/lipophilic-balanced cartridge to extract **75** from the media. Comparison of MT^−^ cells media, previously conditioned with a medium of MT^+^ cells below the SST, and non-conditioned cells by ultra-UHPLC/mass spectrometry showed that **75** was among the MT^−^ cells metabolites as the most significantly upregulated compound. The pheromone was isolated by preparative HPLC, and its MS data, including electron-impact MS fragmentation and MS/MS spectra, suggested it has a diketopiperazine di-L-proline structure (so-called diproline). Its structure was proposed by comparison of chromatographic behavior at co-injection with the standard diketopiperazine (**75**), synthesized from L-proline, as well as by comparison of their CD spectra. Both isolated and synthesized compounds activated Mt^+^ cells, which can be attracted to the diproline source at concentrations of 2 pmol/mg on the bead of SPE sorbent. As a result of the action of pheromones on mature Mt^−^ and Mt^+^ cells, they paired, followed by meiosis, gametogenesis, formation of zygotes, and elongation to the initial cell size and size restoration as a result of the switch to sexual reproduction. The structure of **75** is unprecedented and quite different when compared with other pheromones of any other origin [114].

The activities of nine different diketopiperazine derivatives in attraction bioassay were examined. The pheromone **75** and a pipecolic acid derived diketopiperazine demonstrated the highest attractivities. Surprisingly, the synthetic D-diproline showed a similar attractive action, probably due to the electronic properties of the signal compound being more important than the geometric properties [115,116,117].

On the contrary, with **75**, Sip^+^ and Sip^−^ act on the first stages of signal transduction in diatoms. One of these pheromones, namely SIP^+^ (**76**) from the same *S. robusta,* induces the switch from mitosis-to-meiosis in the opposite mating type and triggers cell cycle arrest and mate attraction. This exometabolite was identified by comparative metabolomics, purified, and characterized. To identify **76**, comparison of exo-metabolomes of MT^+^ versus MT^−^ cell lines below the SST was carried out, and the pheromone-emitting cultures, demonstrating positive bioassay with attraction pheromone (**75**), were used for isolation of **76** using SPE. Purification of SIP^+^ was achieved by a combination of RP-SPE, anion exchange, and normal-phase LC. The metabolite, showing a pseudomolecular ion peak at m/z 842 ([M-H]^+^ in experiments with negative ionization, was identified as one of the most notable upregulation products. SIP^+^ candidate was characterized using HR MS. Transcriptional studies showed that the glutamate-to-proline pathway is upregulated in the diatoms in response to SIP^+^ treatment. The cell cycle arrest by the lowest doses of this extremely active compound confirmed the existence of a mechanism, increasing chances of mate finding, to release the reproductive process with minimal expenses of materials and energy. SIP^+^ (**76**) and SIP^−^ pheromones were secreted in much lower concentrations than **75**. Consequently, metabolic losses of resources at the action of SIPs are minimized, even more so because their release into the seawater is limited to a very short time [118].

**Figure 7:**
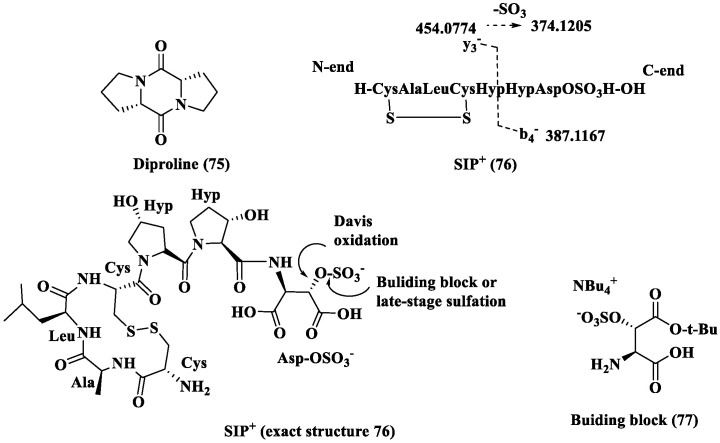
Structures of the diatom *Seminavis robusta* pheromones **75** and **76**.

Seven years after its identification, the large-scale isolation of **76** and subsequent studies led to its structure elucidation by an international team of scientists from Germany, Belgium, and Switzerland, who have overcome the enormous difficulties associated with the extremely low content of this metabolite in the diatom and only episodic appearance in the microalgal cultures during the sexual reproduction stage. The isolation procedure was again started by reversed-phase solid-phase extraction. After it, the obtained extracts were submitted to RP HPLC, followed by studies with positive and negative MS ionizations. HR-ESIMS analysis suggested that **76** with exact [M-H]^−^ mass of 842.2042 (negative mode) is a sulfated metabolite. A minute amount of **76** was obtained from several hundred liters of supernatant from the culture, followed by subsequent isolation and purification, but it was insufficient for NMR analysis. To facilitate the determination of gross formula, the 100% ^15^N isotope enrichment was achieved, when *S. robusta* quantitatively incorporated ^15^N instead of ^14^N after the diatom cultivation in artificial seawater containing ^15^NO_3_ as single nitrogen source. The mass of **76**, fully enriched by ^15^N, was increased by 6.9742, corresponding to seven nitrogen atoms in the studied molecule. Along with other HR MS data, it led to the molecular formula C_29_H_45_N_7_O_16_S_3_. Numerous MS/MS spectra with collision-induced dissociation using different normalized collision energies suggested that **76** contains a sulfate group and consists of two moieties: a linear encompassing a sulfate group and a cyclic, formed by a disulfide bond between two Cys residues. CID-MS fragmentation in the negative mode showed peptide fragmentation with particularly important b_4_ and y_3_ cleavages of the peptide bond between the acyclic fragment, constructed of sulfooxy aspartic acid and two hydroxy proline residues on the C-terminus, and a disulfide cyclic fragment consisting of two Cys, Ala, and Leu on the N-terminus, allowing for proposing structure **76**; that, however, required NMR confirmation. The structure was confirmed by the reduction in the disulfide bond with dithiothreitol, followed by MS analysis (Figure 7).

To carry out reliable MS assignments and increase the sensitivity of NMR analysis, the above-mentioned international team returned to isotope enrichment experiments. The repeated cultivation of the diatom in 80 L of the ^13^C carbonate medium also containing ^15^N nitrate led to the isolation of the ^13^C, ^15^N-enriched sample of SIP + (less than 200 μg), which made possible the detection of only 10 out of 29 carbons in the 1D ^13^C NMR spectrum. However, detailed 2D-NMR spectra of this sample with the corresponding TOCSY, ^1^H, ^13^C HMBC, and ^1^H, ^15^N HMBC experiments in D_2_O provided the crucial information to establish its structure as a heptapeptide (Figure 7). The absolute configuration of 10 stereogenic centers in **75** was determined using the known Marfey’s analysis, often applied to cyclic and other unusual peptides. This analysis showed that all the amino acids in **76** have L-configurations. Hydroxy prolines (Hyps) were assigned as *trans*-4-hydroxy-L-prolines (Hyp) and hydroxy-Asp as L-threo-β-hydroxyaspartic acid. Thus, three out of seven amino acids of this heptapeptide have unusual structures: two Hyp and an unprecedented Asp-OSO_3_^−^. The pheromone (**76**) was proved to be extremely potent, because it induces the production of attraction pheromone **75** in the mating partner at as low as femtomolar concentrations [118].

Finally, this structure was confirmed by the total solid-phase peptide synthesis using two approaches: (i) either via the use of a specially obtained sulfated building block (**77**) on terminal stages of synthesis, or (ii) via Davis oxidation of semi-finished intermediate product of the synthesis, followed by late-stage sulfation of DMF-SO_3_ in terminal amino acid residue at the C-end of **76**. Comparison of NMR spectra of natural and synthetic pheromones was complicated by a significant variability of 1H NMR spectra of both terminal residues of the synthetic product at different pH values. Nevertheless, a conclusion was reached about the identity of these compounds. Synthesis has allowed obtaining a sufficient amount of **76** for further studies of its activity and establishing the structure–activity relationship in a row of its analogs [119].

Thus, a new research field, directed to the discovery and structure elucidation of the first sex pheromones from diatoms, was opened as a result of almost twenty years of very complicated and difficult studies, which could be considered as a significant contribution to chemical ecology and the chemistry of natural products. As it is known, most living organisms, especially those in aquatic environments (90% of the Earth’s habitable area), use chemical signaling, including pheromones, to communicate with each other [120].

It is still unknown whether all diatom taxa possess pheromone systems similar to those recently discovered, or whether they use other pheromones regulating their sexual processes.

The first information on pheromones guiding mating in the diatom *Cylindrotheca closterium* was obtained. To observe the peculiarities of movement of its two mating types, a new capillary bioassay was elaborated. The behavior of these cell lines and the pheromone system of *C. closterium* showed unique complexity, but included some aspects observed in *S. robusta* [121]. The planktonic pennate diatom *Pseudo-nitschia multistriata* also possibly uses a similar signaling system, with recently discovered pheromones, arresting both mating types in the G1 phase, and a sex determining gene *MRP3*, expressed exclusively in MT^+^ strains in a monoallelic manner, was studied. However, nothing is known about the chemical nature and structures of the corresponding pheromones [122]. The increased attention to the studies on molecular mechanisms of sexual processes in diatoms allows hope for the emergence of new breakthrough results in this field in the near future.

Thus, it has been shown that diatoms have signaling systems that ensure successful reproduction in the marine environment by releasing extremely small concentrations of highly active signaling compounds with unique structures. These pheromones have no analogs in any other group of organisms.

### 3.2. Toxins

Domoic acid (**78**, DA) is a potent marine excitotoxin (ME) [123] and natural agonist of glutamate receptors, first isolated from the red macroalgae *Chondria armata* in 1959. Preparations obtained from this species were used for centuries as an anthelmintic in Japan. In other countries, this natural product became known as amnesic toxin after 1987, when shellfish poisoning was indicated in Prince Edward Islands (Canada), resulting in three deaths, unexpectedly caused by DA from the diatom *Pseudo-nitzschia multiseries*. In humans, DA may induce a potentially lethal syndrome with short-term memory loss [124]. The toxin is transferred via food webs from *Pseudo-nitzschia* cells to consumers of diatoms that led to the tremendous loss of cultivated mollusks and mass death of marine animals, including sea lions and whales as well as sea birds. Later, about half of *Pseudo-nitzschia* spp. were shown to produce DA [125].

**Figure 8:**
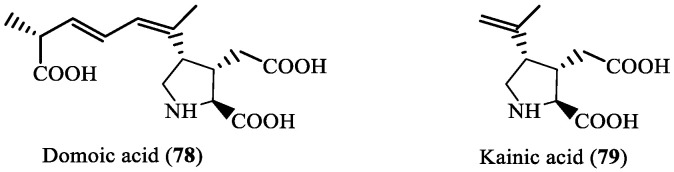
Some neurotoxins of algae.

Kainic acid (**79**, KA) is a related ME found in another red macrophyte, *Diginea simplex*, which for many years has been used in neuropharmacology as a reagent, stimulating the so-called kainate receptors in the brain (Figure 8). The action of both these toxins on higher animals and humans is determined by the capability of DA and KA to overstimulate the central nervous system by binding to the glutamate receptors. DA is a more potent stimulator of glutamate receptors in comparison with KA.

Long-term studies on DA are concerned with several problems, such as determining why some species of diatoms produce this toxin only occasionally. It is poorly understood what regulates DA expression. Another problem is related to the origin and evolution of the biosynthesis of DA, which was found in two distant taxa: red macrophytes and diatoms. Finally, one more problem is connected with the interactions of DA with other marine organisms in environment.

The recent largest harmful algal bloom, dominated by the toxic diatom *P. australis*, occurred in the Northeast Pacific in 2015, led to a loss of USD 100 million both in fisheries and as a result of the death of sea mammals. Fifty-two near-weekly phytoplankton samples were collected at a bloom hotspot in Monterey Bay and analyzed for transcription of known DA biosynthesis (dab) genes as the primary origin of toxicity [126].

Exclusively important data have been obtained in the last decade concerning the biosyntheses of excitory toxins (**78** and **79**) in diatoms. The biosynthesis of domoic acid (**78**) was shown to be encoded by a specific four-gene cluster (dab genes), consisting of terpene cyclase (dabA), a hypothetical protein (dabB), dioxygenase (dabC), and CYP450 (dabD). Transcriptome sequencing of this cluster from the diatom *Pseudo-nitzschia multiserries* was implemented by Brunson et al. [127] in order to identify and co-localize dab genes in this cluster.

**Scheme 7:**
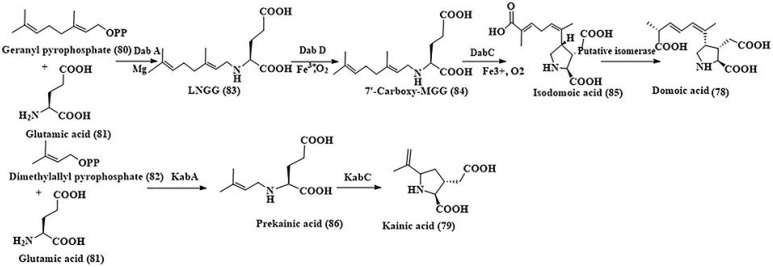
Biosynthesis of domoic and kainic acid (adapted from [127,128]). Dab – domoic acid biosynthesis; Kab – kainic acid biosynthesis; L-NGG – N-geranyl-L-glutamic acid; 7’Carboxy-NGG – 7’carboxy-N- geranylglutamic acid.

Two-enzyme pathway of kainic acid (**79**) biosynthesis from L-glutamic acid (81) and dimethylallyl pyrophosphate (**82**) was discovered in the red macrophytes *Digenea simplex* and *Palmaria palmata*, and the corresponding genes were co-clustered in red algal DA (*rad*) biosynthetic gene cluster in their genomes. In addition, a key α-ketoglutarate-dependent dioxygenase was used to develop a biotransformation method for biotechnological producing of **79** on a gram scale; the scheme of these biosyntheses (Scheme 7) is given below [128].

Recently, the 507 Mb genome of the brown alga *Chondria armata* was sequenced, and several copies of the red algal DA (*rad*) biosynthetic gene cluster were found. The *rad* genes were organized similarly to the diatom DA biosynthesis cluster in terms of gene synteny. This work suggested a complex evolutionary history for DA biosynthesis, involving gene transfers and neofunctionalization [129].

Diatoms and red algae have been found to share a similar sequence of conversions during the biosynthesis of domoic acid with CYP450-catalyzed oxidation to carboxylic acid occurring before cyclization in both organisms [130]. It is assumed that a similar biosynthetic reaction to install carboxylic acid on the scaffold DA developed independently in diatoms and red algae [129].

At the same time, there are still gaps in our understanding of the enzymology underlying DA biosynthesis. In particular, the responsible isomerase in the process of DA biosynthesis remains unknown [127], which suggests further studies of candidate genes, as well as the activity of CYP450 DabD and other enzymes.

Domoic acid chemically interacts with other marine organisms during diatom blooms, as discussed in the recent review by Kuhlisch et al. [131]. It inhibits the growth of the diatom *S. marinoi*, which does not produce DA, while diatom cells, containing DA, are rejected by some copepods [130]. Actually, the toxicity of *P. seriata* grew in the presence of copepods without contact between producers of the toxins and these small crustacean grazers feeding on toxic diatoms, probably due to the chemical cues between them [131]. The recent genetic study showed that genes involved in signal transduction are probably induced in *Pseudo-nitzschia* cells when they receive unknown signals from copepods [132]. Nevertheless, until now, the ecological role of DA in marine ecosystems remains insufficiently clear.

## 4. Conclusions

Over the past decade, a substantial volume of new information has been obtained on the biologically and ecologically active metabolites of diatoms, so that it has become impossible to discuss all new, important, and interesting discoveries within a single review article. Consequently, this review is limited to a selection of specific classes of these marine natural compounds.

During the last two decades, the focus of molecular studies on diatoms has shifted from the identification and structure determination of new metabolites and studies on their potential applications to the unraveling of biosyntheses and identification of enzymes and enzymatic clusters, which orchestrate the formation of natural products in diatoms, as well as to a deeper understanding of their biological roles and activities. The completion of whole genome sequencing for several diatom species, combined with the application of molecular engineering and synthetic biology methods, has enabled progress in addressing key issues concerning the structural diversity and functions of diatom metabolites such as marine oxylipins, pheromones, and glycolipids. Several important applied achievements concerned preparation of valuable fatty acids, triacylglycerols, glycolipids, and other physiologically active substances of diatoms.

The identification and structure elucidation of pheromones in diatoms is, in our opinion, one of the significant recent successes of marine ecology and chemistry of marine natural compounds. The discovery of prostaglandins in diatoms and the analysis of their fractions will allow a better understanding of the origin and evolution of this group of natural products important for medicine.

Over the past two decades, interest has grown rapidly in the industrial production of valuable metabolites from diatoms using modern biotechnological approaches, as well as in utilizing their chemical components for biofuels, pharmaceuticals, and preventive healthcare agents. As a result, new approaches have been developed to significantly increase the yield of valuable products.

Some studies, the results of which were partly discussed in the review, also belong to a new rapidly developing scientific field, such as the search for new infochemicals and signal metabolites, chemically regulating alga–bacterium, alga–alga, alga–parasite, alga–grazer, and others’ interactions. Such investigations hold an untapped potential of new important discoveries.

## Figures and Tables

**Table 1:** Some recently studied chrysolaminarins from diatom microalgae.

Species	MW (kDa)	Branching	Yeld (%)	Refs
*Phaeodactylum tricornutum*	10	β-1,6-branching	>14	[23]
*Odontella aurita*	7.75	β-1,6-branching	48.1	[23,58]
*Skeletonema costatum*	6–13	β-1,6-branching and β-1,2-branchings	32	[52]
*Staurontis amphioxys*	4	β-1,6-branching and β-1,2-branchings	nd	[52]
*Pinnularia viridis*	>10	β-1,6-branching and β-1,2-branchings	nd	[52]
*Stephanodiscus meueri*	40	β-1,6-branching	0.5	[52]
*Stephanodiscus meueri*	2–6	β-1,6-branching	0.6	[52]
*Thalassiosira weissflogii*	5–13	No branching	nd	[52]
*Chaetoceros debilis*	4.9	Highly branching, β-1,6-branching	10	[52]
*Conticribra weissflogii*	11.7	Low proportion of β-1,6-branchings	29.9	[60]

nd—not determined.

## Data Availability

All data used in this study are available upon request from the corresponding author.
